# Development and Prospect of Esophageal Tissue Engineering

**DOI:** 10.3389/fbioe.2022.853193

**Published:** 2022-02-17

**Authors:** Rui Xu, Xinnan Fang, Shengqian Wu, Yiyin Wang, Yi Zhong, Ruixia Hou, Libing Zhang, Lei Shao, Qian Pang, Jian Zhang, Xiang Cui, Rongyue Zuo, Liwei Yao, Yabin Zhu

**Affiliations:** ^1^ The Affiliated Hospital of Medical School, Ningbo University, Ningbo, China; ^2^ School of Medicine, Ningbo University, Ningbo, China; ^3^ Ningbo Institute of Materials Technology and Engineering, Chinese Academy of Sciences, Ningbo, China; ^4^ The Affiliated Lihuili Hospital, Ningbo University, Ningbo, China

**Keywords:** esophageal repair, tissue engineering, single-layer scaffold, multi-layer scaffold, stem cells

## Abstract

Currently, patients with esophageal cancer, especially advanced patients, usually use autologous tissue for esophageal alternative therapy. However, an alternative therapy is often accompanied by serious complications such as ischemia and leakage, which seriously affect the prognosis of patients. Tissue engineering has been widely studied as one of the ideal methods for the treatment of esophageal cancer. In view of the complex multi-layer structure of the natural esophagus, how to use the tissue engineering method to design the scaffold with structure and function matching with the natural tissue is the principle that the tissue engineering method must follow. This article will analyze and summarize the construction methods, with or without cells, and repair effects of single-layer scaffold and multi-layer scaffold. Especially in the repair of full-thickness and circumferential esophageal defects, the flexible design method and the binding force between the layers of the scaffold are very important. In short, esophageal tissue engineering technology has broad prospects and plays a more and more important role in the treatment of esophageal diseases.

## Introduction

**FIGURE 1:**
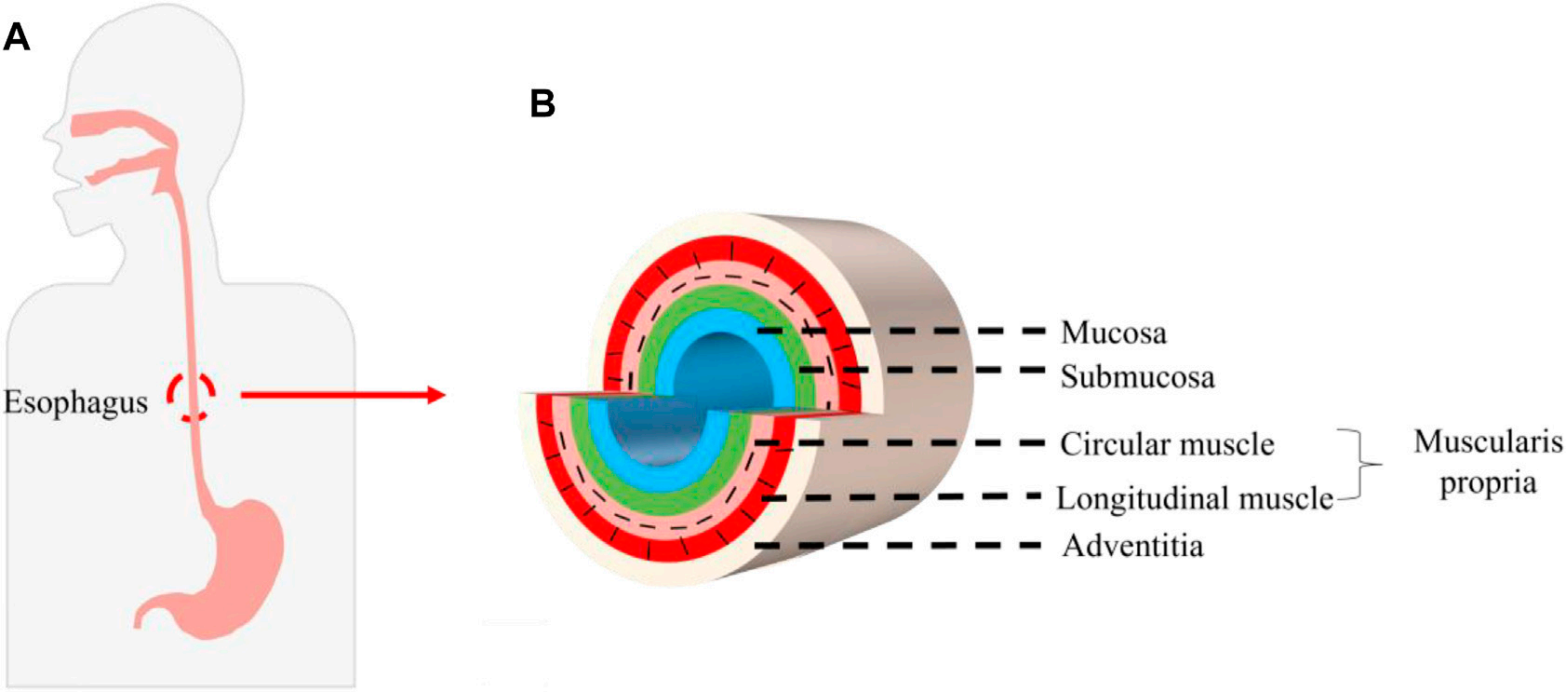
Structure of the human esophagus. **(A)** Position of the esophagus in the human body. **(B)** Schematic diagram of cross section of the esophagus.

Esophageal cancer is the seventh most common cancer in the world and ranks sixth in the world in terms of lethality among all malignant tumors. Of the 500,000 new cases worldwide each year, about half occur in China. It is the fifth most commonly diagnosed cancer and the fourth leading cause of cancer death in China. The fatality rate of esophageal cancer remains high, which seriously affects people’s lives and health (Pennathur et al., 2013; Chen et al., 2016; Chang et al., 2017; Huang et al., 2018; Uhlenhopp et al., 2020). Surgical alternative therapy requires replacement of the stomach, jejunum, colon, and other autologous tissues, but replacement is likely to cause high morbidity and mortality, and at the cost of normal tissue damage, it seriously affects the quality of life (Dua et al., 2016; Arakelian et al., 2018). In recent years, tissue engineering technology has been used to construct bionic esophageal scaffolds, which avoids taking materials from patients, reduces the risk of high-risk surgery and high mortality and morbidity caused by postoperative surgery, and provides a new method for esophageal repair and reconstruction (Zhu et al., 2017). The esophagus is composed of the mucosa, submucosa, muscularis propria, and adventitia. The muscularis propria is a multi-layer structure with an inner ring and an outer longitudinal shape, called the circular muscle and the longitudinal muscle. The cells mainly include mucosal epithelial cells (ECs) and smooth muscle cells (SMCs) (Peirlinck et al., 2018; Blank et al., 2019; Farhat et al., 2021) (Figure 1). Therefore, as an ideal esophageal tissue engineering scaffold and a bionic multi-layer structure of the esophagus, it is endowed with corresponding supporting functions for different parts. How to combine each layer of scaffold effectively is one of the important problems that must be considered when constructing a multi-layer scaffold. In view of this, how to design a bionic multi-layer composite scaffold, which has both multi-layer structure and multi-function and ensures the firm connection between each layer, is a scientific problem of great concern in this field and has important scientific significance and potential application value.

## Single-Layer Esophageal Scaffolds

### Scaffold Material

In the research of esophageal tissue engineering, researchers have conducted a large number of innovative research studies on the construction of single-layer scaffolds to repair the mucosal layer or the muscle layer. Single-layer scaffolds are classified according to the choice of materials, mainly including acellular matrix or (and) polymer scaffolds. The acellular matrix includes the small intestinal submucosa (SIS), urinary bladder submucosa (UBS), esophageal mucosa, etc. Polymer materials include polylactide (PLA), poly (l-lactide-co-ε-caprolactone) (PLGA), poly (3-hydroxybutyrate-co-3-hydroxyvalerate) (PHBV), polylactide-poly (ɛ-caprolactone) (PLA-PCL), polyurethane (PU), etc. (Badylak et al., 2000; Nieponice et al., 2014; Kuppan et al., 2016; Tan et al., 2016; Dorati et al., 2017; Kuppan et al., 2017; Luc et al., 2018) (Table 1).

**TABLE 1:** Classification according to materials of single-layer scaffolds.

Author	References	Scaffolds	Formation method	Loading cell	Study	Biota	Outcomes
Badylak et al.	Badylak et al. (2000)	SIS, UBS	Multi-layer esophageal scaffold composed of the ECM	—	Patch and full segmental esophageal implantation	Canine	89% mortality. Complete and confluent squamous epithelium on the surface of the scaffold
Dorati et al.	Luc et al. (2018)	Decellularized esophagus	Matrix of decellularized esophagus	—	Full segmental esophageal implantation	Pig	16% mortality. Complications are reported in the treatment group
Nieponice et al.	Nieponice et al. (2014)	UBS	Multi-layer esophageal scaffold composed of the ECM	—	Patch esophageal implantation	Human	0% mortality. All patients were able to save their esophagus
Kuppan et al.	Kuppan et al. (2017)	PHBV, PCL, gelatin	Aligned nanofibrous scaffold made of PHBV, PHBV-gelatin, PCL, and PCL-gelatin	ECs, SMCs	*In vitro*	—	ECs and SMCs can divide into two different levels
Dorati et al.	Dorati et al. (2017)	PCL, PLA, chitosan	Multi-layer patch	FBCs	*In vitro*	—	Cells grow better on multi-layer patches than on single-layer patches
Tan et al.	Tan et al. (2016)	PLC	Fusion integrated scaffold made of PLC as the material	FBCs	*In vitro*	—	Good mechanical properties and biocompatibility
Lv et al.	Lv et al. (2014)	PCL, SF	Using PCL as the raw material for fiber scaffolding by electrospinning	—	Partial-thickness esophageal implantation and subcutaneous implantation	Rabbit	0% mortality. The esophageal mucosa has regenerated, while the scaffold has been ruptured
Zhu et al.	Zhu et al. (2005)	PLGA, collagen	Using collagen to modify the surface of PLGA	SMCs	*In vitro*	—	Collagen-modified PLGA promotes the growth of SMCs in the esophagus
Zhu et al.	Zhu et al. (2006)	PLLC	Cellulose and collagen modified the PLLC surface	SMCs, ECs, FBCs	*In vitro*	—	PLLC with collagen or cellulose supports the cell attachment, growth, and functional forms
Gong et al.	Gong et al. (2013)	PU	Making a micro-pattern on the surface of PU	SMCs	Partial-thickness esophageal implantation	Rabbit	0% mortality. The regenerative tissue is tightly attached to the surface of the scaffold material
Tam et al.	Tan et al. (2013)	SIS	Single-layer esophageal scaffold	MSCs	Patch and full segmental esophageal implantation	Pig	0% mortality. Transplantation of MSCs-SIS appears to promote epidermalization, vascularization, and muscle regeneration
Marzaro et al.	Marzaro et al. (2020)	Decellularized esophagus	Acellular matrix of esophageal muscle	MSCs	Partial-thickness esophageal implantation	Pig	0% mortality. Half of the unvaccinated cell groups have narrow esophagus

PLC, poly(l-lactide-co-ε-caprolactone); FBCs, fibroblast cells.

From the perspective of single-layer scaffold structure, the classification mainly includes acellular matrix, membranes grafted with biomolecules, electrospinning scaffolds, micro-pattern scaffolds, etc. (Hou et al., 2016; Wei et al., 2018; Zhuravleva et al., 2019; Chaitin et al., 2021; Levenson et al., 2021) (Table 2). In order to accurately simulate the inner ring and outer longitudinal structure of the muscle layer, electrospinning scaffolds and micro-pattern scaffolds are mainly used (Gong et al., 2013; Kuppan et al., 2017). Our team has conducted a large number of *in vitro* and *in vivo* animal studies on single-layer scaffolds. The original idea was to graft collagen on the surface of polymer materials such as PLGA and poly (l-lactide-co-caprolactone) (PLLC) to improve their biocompatibility (Zhu et al., 2005; Zhu et al., 2006). Then, the polycaprolactone/silk fibroin (PCL/SF) electrospinning scaffold is made by electrospinning technology, and the electrospinning fiber pore size can simulate the structure of the extracellular matrix (ECM) (Lv et al., 2014). At this stage, the micro-pattern membrane technology is used to construct a new type of esophageal bionic scaffold, which has successfully constructed a micro-pattern PU scaffold and double-layer scaffold of the esophageal acellular matrix (Hou et al., 2019; Wang X. et al., 2020).

**TABLE 2:** Classification according to the construction of single-layer scaffolds.

Author	References	Scaffolds	Formation method	Loading cell	Study	Biota	Outcomes
Wei et al.	Wei et al. (2018)	CPU	SF-modified CPU surface	MSCs	*In vitro*	—	SF can enhance the interaction between cells and the biocompatibility of the material
Paolo et al.	Zhuravleva et al. (2019)	Polyamide-6	Polyamide-6 electrospinning scaffold	HUVEC, MSCs	*In vitro*	—	The electrospinning structure can simulate the acellular structure of the esophagus
Hou et al.	Hou et al. (2016)	PU	PU scaffold with a micro-pattern surface	—	Partial-thickness esophageal implantation	Rabbit	0% mortality. The new muscle layer grows in the direction of the micro-pattern channel
Kang et al.	Chaitin et al. (2021)	Esophagus	Matrix of the decellularized esophagus	ESCCs, FBCs	*In vitro*	—	The co-culture of FBS and ESCC could secrete more endometrialin

CPU, poly(ester urethane); HUVEC, human umbilical vein endothelial cell; ESCCs, esophageal squamous cell carcinomas.

### Cell Seeding

**FIGURE 2:**
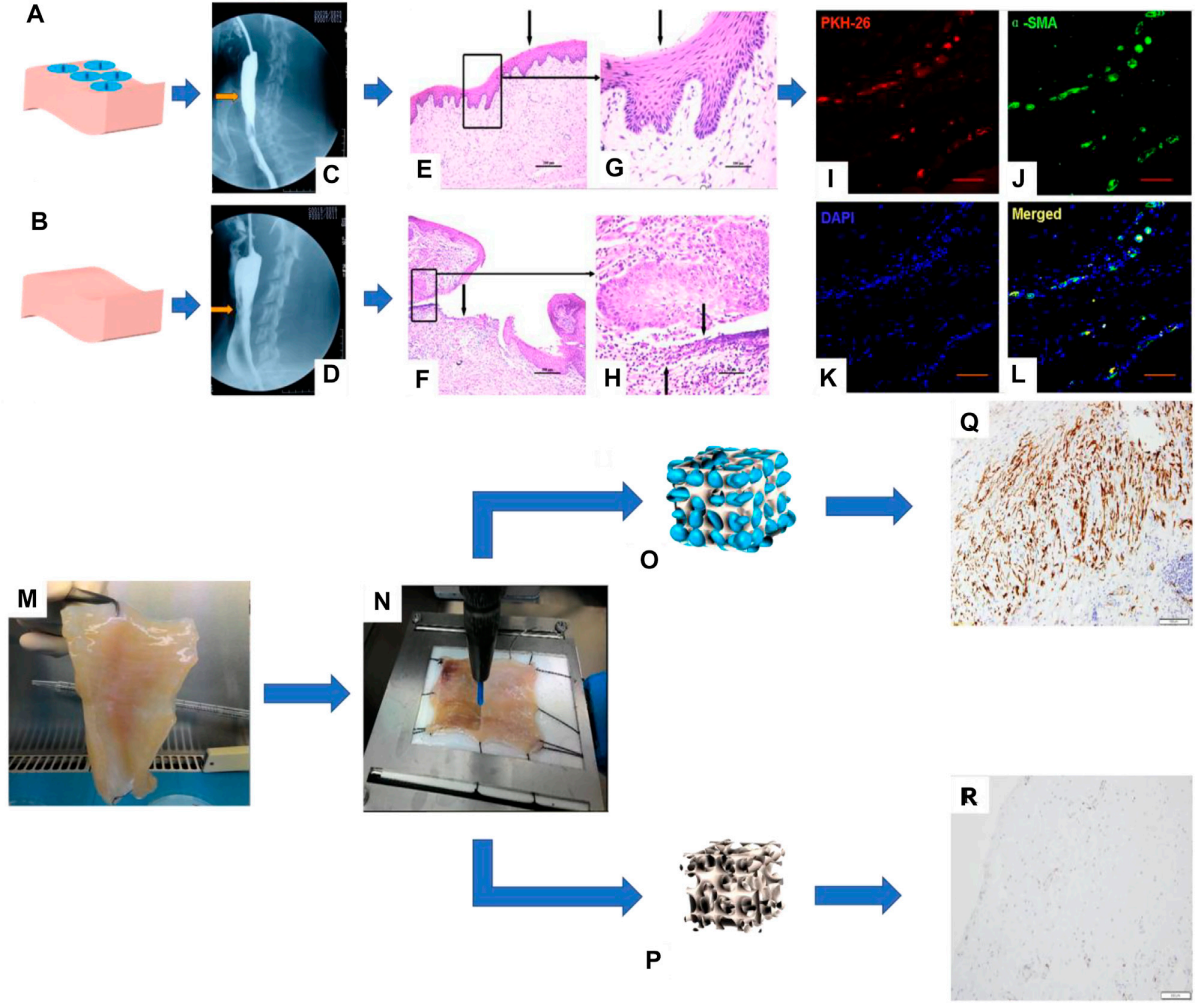
I. Repair of a canine esophageal defect with SIS combined with MSCs. **(A)** SIS + MSCs. **(B)** Simple SIS. **(C, D)** Barium esophagus examination. **(E, F)** H&E staining of canine esophagus tissue and **(G, H)** its partial magnification. **(I–L)** Immunofluorescence staining to detect the expression of living cell marker proteins PKH-26 and *α*-SMA in canine esophagus tissue (Tan et al., 2013). II. Esophageal muscular acellular matrix repairs porcine esophageal defects: **(K)** Porcine esophageal muscular acellular matrix. **(L)** Microscopic perforation treatment. **(M, N)** Schematic diagram of MSC growth in the acellular matrix scaffold. **(O, P)** Immunohistochemical staining to detect the expression of actin and desmin in porcine esophagus tissue (Marzaro et al., 2020).

The abovementioned studies carried out the structural bionic design of single-layer scaffolds from the perspectives of material selection and construction methods and achieved a series of achievements in the damage repair of the esophageal mucosa and muscularis. Studies have shown that if cells are introduced into scaffolds, they will work synergistically with scaffolds in the microenvironment *in vivo* to further enhance functional repair of tissues. For example, Badylak et al. made artificial defects in the dog’s esophagus and used ECM derived from SIS or UBS to repair the esophageal defect. After 35 days, the scaffold was partially covered by the squamous epithelium, and only scattered skeletal muscle cells surrounded collagen connective tissue (Badylak et al., 2000). Xie et al. proved that, after implantation in dogs, SIS alone is not completely endothelialized. When bone marrow mesenchymal stem cells (MSCs) are combined with SIS, the results show that the defect site is completely endothelialized, the muscle layer is regenerated, and the new microvessels are dense (Tan et al., 2013) (Figure 2).

**FIGURE 3:**
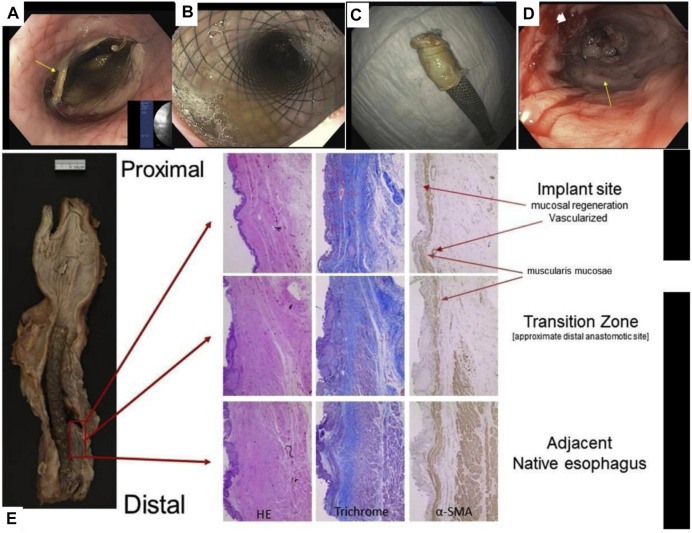
PU combined with MSCs for reconstruction of the human esophagus. **(A)** EGD image of the esophagus after scaffold placement. **(B)** EGD image of the esophagus after scaffold deployment. **(C)** The removed CEI scaffold assembly is adhered to the scaffold. **(D)** EGD image of esophageal neoplastic tissue. **(E)** Histological examination of esophageal sections, including H&E staining, Masson staining, and immunohistochemical staining to detect the expression of *α*-SMA (Aho et al., 2021).

Therefore, researchers have introduced cells, such as ECs, SMCs, and stem cells, into biomimetic single-layer scaffolds to construct tissue engineering scaffolds. The ECs or SMCs are difficult to be widely used due to the large damage to the donor when they are acquired and the limited ability of proliferation and differentiation after cell expansion. On the contrary, stem cells have the advantages of large differentiation potential, strong proliferation ability, convenient and easy acquisition from the body, and the ability to differentiate into specific cells in tissue. So, they are widely used as seed cells in the field of tissue engineering. For example, Ivo et al. combined MSCs and decellularized esophageal muscle tissue to repair the esophagus *in situ* in pigs, which showed new muscle tissue compared with the decellularized esophageal muscle layer alone (Marzaro et al., 2020) (Figure 2). Aho et al. Using PU material, autologous adipose-derived mesenchymal stem cells were seeded to form a cell-span esophageal implant (CEI). After resection of the patient’s esophageal cancer, *in situ* repair was performed using CEI and followed by esophagogastroduodenoscopy (EGD). After the patient’s death, histological examination revealed esophageal luminal epithelialization and partial muscle regeneration 7.5 months after scaffold implantation (Aho et al., 2021) (Figure 3).

Therefore, single-layer bionic scaffold combined with stem cells to repair the mucosal layer or muscle layer is the current main research direction. On the basis of mucosal layer or muscle layer repair, higher clinical requirements for esophageal repair are proposed, such as full-thickness or circumferential defect, which requires simultaneous repair of the mucosal layer, submucosal layer, and muscle layer of the esophagus. Therefore, it is particularly important to design a multi-layer functional bionic scaffold.

## Multi-Layer Esophageal Scaffolds

### Construction Method of Scaffolds

Researchers have studied full-thickness or circumferential defects by designing lamellar or tubular bionic scaffolds. According to the research methods, it is mainly divided into two categories: scaffolds and scaffolds/cells composite. The scaffolds were prepared by the one-step method or multi-step method. The one-step method is to mix the scaffold material into a whole through melting, electrostatic spinning, temperature-induced sedimentation, etc., and different components complement each other and work in synergy. For example, Tan et al. melted PCL/PLA and stretched it into a directional spinning tubular structure (Tan et al., 2016). The multi-step method is based on the perspective of esophageal structure bionics, combining different layers of scaffold materials through a certain link method. Joshua et al. prepared the silk fibroin double-layer scaffold by solution pouring (Gundogdu et al., 2021). Rossella et al. used electrospinning and temperature-induced sedimentation to construct two double-layer scaffolds (Pisani et al., 2020). Saverio et al. designed a PU electrospinning three-layer scaffold (inner and outer layer pore diameter>10 μm; middle layer <10 μm) (Soliman et al., 2019) (Table 3).

**TABLE 3:** Classification according to the construction of bionic scaffolds.

Author	References	Scaffolds	Formation method	Loading cell	Study	Biota	Outcomes
Joshua et al.	Gundogdu et al. (2021)	SF	Bilayer silk fibroin	—	Partial-thickness esophageal implantation	Pig	0% mortality. Scaffold shifts, esophageal stenosis, and other complications were seen
Rossella et al.	Pisani et al. (2020)	PLA, PCL	Temperature-induced settlement double-layer scaffold, electrospinning double-layer scaffold	MSCs	*In vitro*	—	Scaffolds constructed in two ways are suitable for esophageal regeneration
Saverio et al.	Soliman et al. (2019)	PU	Three-layer bracket	MSCs, SMCs	*In vitro*	—	Cells can survive on three layers of scaffold and be separated by the middle layer

### Study on Scaffolds/Cells

Through the abovementioned research and analysis, it can be seen that the design of multi-layer scaffolds is the guiding ideology of bionics, but these scaffolds still cannot completely induce the structural growth of tissues. Therefore, researchers have constructed scaffolds with cells to enhance the repair function of tissue. Natural materials such as esophageal acellular matrix, SIS, and collagen scaffold are compounded with cells.

**FIGURE 4:**
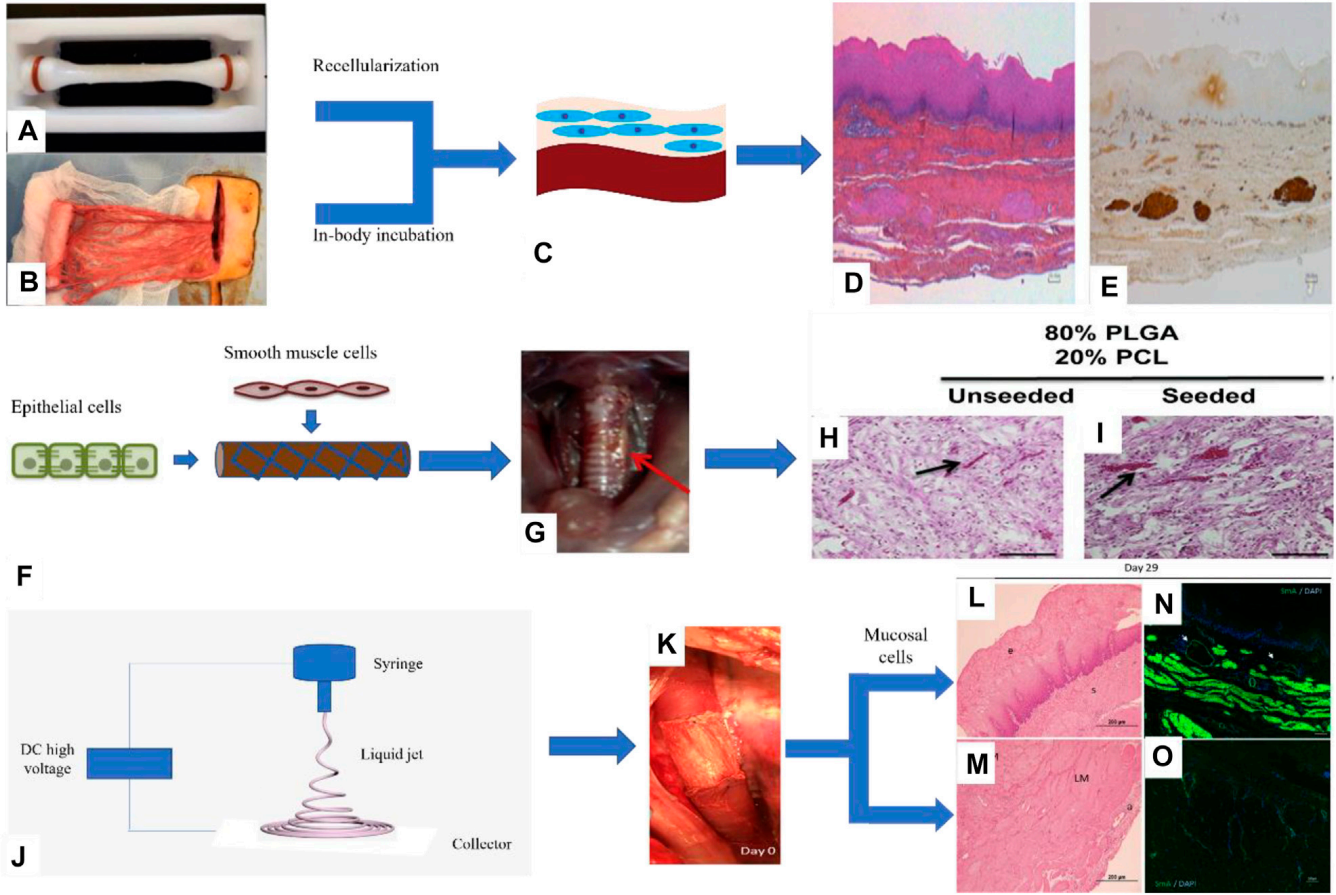
Porcine esophageal acellular matrix and omentum construct a double-layer scaffold to repair esophageal defects. **(A)** Acellular matrix. **(B)** Decellularized matrix composite omentum. **(C)** Omentum maturation in pigs. **(D)** H&E staining of pig esophagus sections. **(E)** Immunohistochemical staining to detect the expression of desmin in porcine esophagus tissue (Levenson et al., 2021). II. The PLGA/PCL electrospinning scaffold was prepared by the one-step method combined with ECs and myocytes to repair esophageal defects: **(F)** Cells were cultured on the inner and outer surfaces of the scaffold. **(G)** The scaffold is a patch to repair esophageal injury in rats. **(H, I)** H&E staining of rat esophagus sections (Jensen et al., 2015). III. PU electrospun scaffolds combined with mucosal cells to construct composite scaffolds involved in porcine esophagus reconstruction: **(J)** Schematic diagram of electrospinning. **(K)** Composite scaffold for *in situ* replacement of the esophagus. **(L, M)** H&E staining of porcine esophagus sections. **(N, O)** Immunofluorescence staining was used to detect the expression of *α*-SMA in porcine esophagus tissue (Barron et al., 2018).

Guillaume et al. designed the esophageal mucosal acellular matrix/omentum double-layer scaffold, in which MSCs were cultured on the acellular matrix, and the omentum re-matured in pigs. As a result, it was found that 3 months after the esophageal replacement surgery, a new epithelium and muscle regeneration were visible (Levenson et al., 2021) (Figure 4). Paola et al. used the method of organoid culture to construct a multi-layer esophageal scaffold with cells *in vitro*. The researchers re-seeded ECs on the acellular matrix of the rat esophageal mucosa and allowed the cells to grow in the lumen of the acellular scaffold to construct the esophageal mucosal layer and co-cultured human or mouse fibroblasts and mouse neural crest cells *in vitro*. The muscle layer is constructed and then implanted into the rat omentum for *in vivo* culture to promote angiogenesis and build a multi-layer esophageal structure together with the mucosal layer. This kind of esophageal tissue composed of cells is more complete than the commonly used acellular matrix and other natural materials, but it needs to be verified by animal experiments to prove its positive significance (Urbani et al., 2018) (Figure 4).

In addition to natural materials, synthetic polymer materials such as PLGA, PCL, and PU have also been studied in combination with cells. For example, Christine et al. prepared a PLGA/PCL electrospun tubular esophageal scaffold, the inner cavity of the scaffold was compounded with autologous ECs, and the outer side was compounded with autologous SMCs. The composite scaffolds containing cells were cultured in an *in vitro* bioreactor for a period of time and then implanted into the mouse esophagus *in situ*, the esophagus is still viable after 2 weeks, and the cells maintain the phenotype (Jensen et al., 2015) (Figure 4). Dennis et al. combined porcine esophageal mucosal cells and electrospun PU scaffold into a tubular scaffold and implanted it into the whole-peripheral defect of the porcine esophagus, and the results showed that the mucosal layer, submucosa, and muscle layer of the esophagus regenerate simultaneously and have abundant blood vessels (Barron et al., 2018) (Figure 4).

**FIGURE 5:**
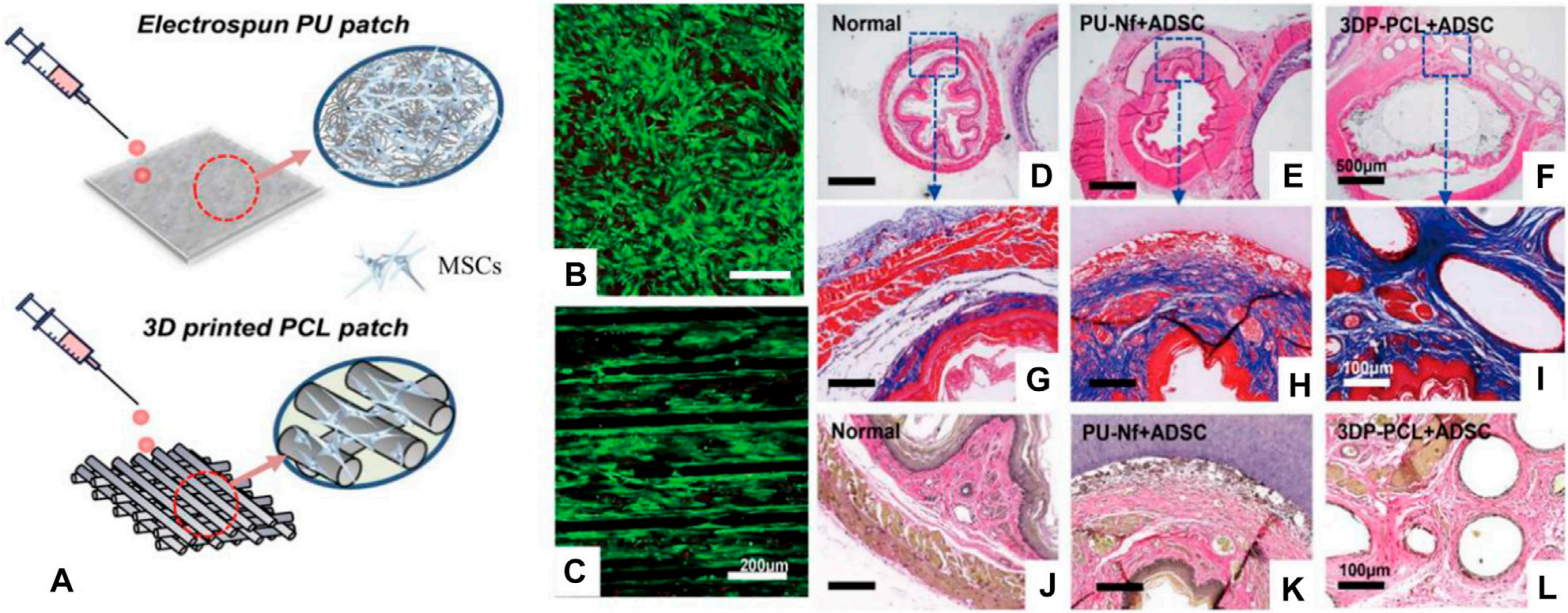
3D printed PCL scaffold and electrospun PU scaffold combined with MSCs to repair the esophageal defect. **(A)** Schematic diagram of the 3D printed PCL scaffold and electrospun PU scaffold. **(B,C)** The live/dead cell assay on the scaffold surface was studied. **(D–F)** H&E staining of rat esophagus sections. **(G–I)** Masson staining of rat esophagus sections. **(J–L)** Elastic fiber staining of rat esophagus sections (Park et al., 2021).

Different from the traditional scaffolds, 3D printing scaffolds have many advantages, such as the flexibility of preparation methods, the customization of irregular tissue damage parts, and the ability to prepare scaffolds with very complex structures (Memic et al., 2017; Matai et al., 2020; Wan et al., 2020; Barros et al., 2021; Wu et al., 2021; Yang et al., 2021). The 3D printing scaffolds have been studied in esophageal repair. For example, Chung et al. used a 3D melt extrusion method to construct a polycaprolactone (PCL) 3D printing scaffold, seeded MSCs on the scaffold to participate in esophageal reconstruction, cells grew along the direction of the scaffold, and implanted it in the defect of the rat esophagus. The results show that the new tissue repaired by the 3D printing scaffold is similar to natural tissue and has obvious advantages compared with electrospun PU scaffolds (Figure 5) (Park et al., 2021). Although 3D printing scaffolds have many advantages, this method also has its own limitations, such as lack of diversity of bio-ink, harsh printing conditions (high temperature or UV curing), and expensive equipment for printing cells.

The mixed use of polymer synthetic materials and natural materials has gradually become the focus of research. For example, Jonathan et al. used electrospinning technology to make PLGA fiber layers on the SIS acellular matrix to form a double-layer esophageal scaffold, the results showed that human esophageal smooth muscle cell culture experiments and subcutaneous embedding presented good biocompatibility (Syed et al., 2019), but further research is needed for *in vivo* repair.

**FIGURE 6:**
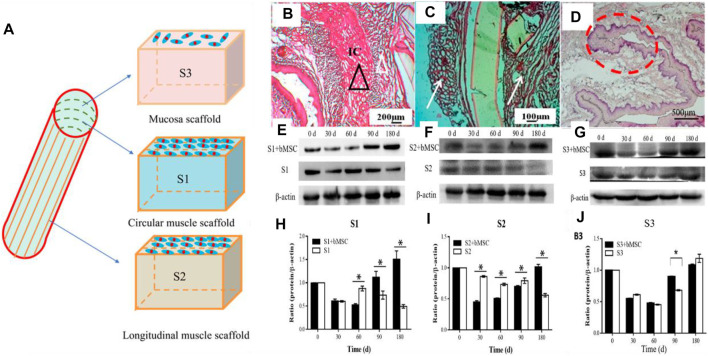
Multi-layer esophageal scaffold combined with stem cells to repair esophageal defect in rabbits. **(A)** Schematic diagram of the three-layer scaffold; S1, S2, and S3, respectively, represent the inner ring muscle, outer longitudinal muscle, and mucosal layer of the esophagus. **(B–D)** H&E staining of rabbit esophagus sections at 180 days **(E–G)** Western blot evaluated the expression of *α*-SMA (S1, S2) and CK-14 (S3), respectively, in rabbit esophageal defect. **(H–J)** Quantitative calculation of (E–G) using ImageJ software (*n* = 3). *p < 0.05 (Wang X. et al., 2020).

Our group’s previous study used micro-pattern technology to construct a three-layer scaffold, which corresponds to the inner ring muscle (S1), outer longitudinal muscle (S2), and mucosal layer (S3) of the esophagus. After inoculating MSCs on the composite scaffold, it was implanted into the esophageal defect. The results showed that there was new esophageal tissue, including the muscle layer and mucosal layer. However, the PU material can still be found in the tissue 180 days after implantation, which may affect the speed of muscle regeneration (Wang X. et al., 2020) (Figure 6).

## Problems and Challenges

In conclusion, although studies on the repair of the full-thickness or circumferential defect of the esophagus have achieved many tentative results, the following key issues still need to be further developed and improved, in order to obtain a bionic scaffold that is closer to the natural esophageal structure and function, so as to be used in the clinical treatment of esophageal cancer patients as soon as possible and benefit mankind.

### Precision Bionics

At present, there are two main construction methods of esophageal scaffold, one-step construction and multi-step construction. The advantage of one-step construction is that it is relatively simple, only need one or more types of biomaterials are required, and generally, the binding force between the support layers is strong and stable. However, the disadvantage is that the material and function are relatively single, so it is very difficult to accurately simulate the multi-layer structure of the esophagus and give specific functions to each layer of the scaffold. Since the multi-layer scaffold prepared by the multi-step construction method is flexible, it can provide different materials (natural materials, synthetic materials, or both) and cells (ECs, SMCs, or stem cells) for each layer of the scaffold, so as to more accurately mimic the structure and function of the esophagus. Therefore, it is the current development direction to prepare an accurate bionic multi-layer esophageal scaffold by multi-step construction.

### The Firmness Between Multi-Layer Esophageal Scaffolds

For multi-layer scaffolds prepared by multi-step construction, the firmness between the scaffolds is another key issue. If the adhesion between scaffolds is poor, the multi-layer scaffolds will fall off or shift, which will seriously affect the repair effect of the esophagus. The connection modes between the layers of multi-layer scaffolds include the solution casting method (Gundogdu et al., 2021), temperature-induced precipitation method (Dorati et al., 2017), solvent volatilization method of electrospinning (Chung et al., 2015), and glue bonding (Deng et al., 2019). The first three methods are not universally applicable because solutions or solvents may dissolve the active components such as protein, growth factor in the scaffold, and too high or low temperature is not conducive to the introduction of proteins and cells into the scaffold. Glue bonding does not affect the design of each layer of the scaffold, as long as the scaffold prepared separately is combined, which is a simple combination method with universal applicability. Generally, it is relatively easy for the glue to adhere to objects in a dry environment, but it remains a great challenge for repair in a wet environment (exudate or blood at the injury) or dynamic adhesion (human movement).

**FIGURE 7:**
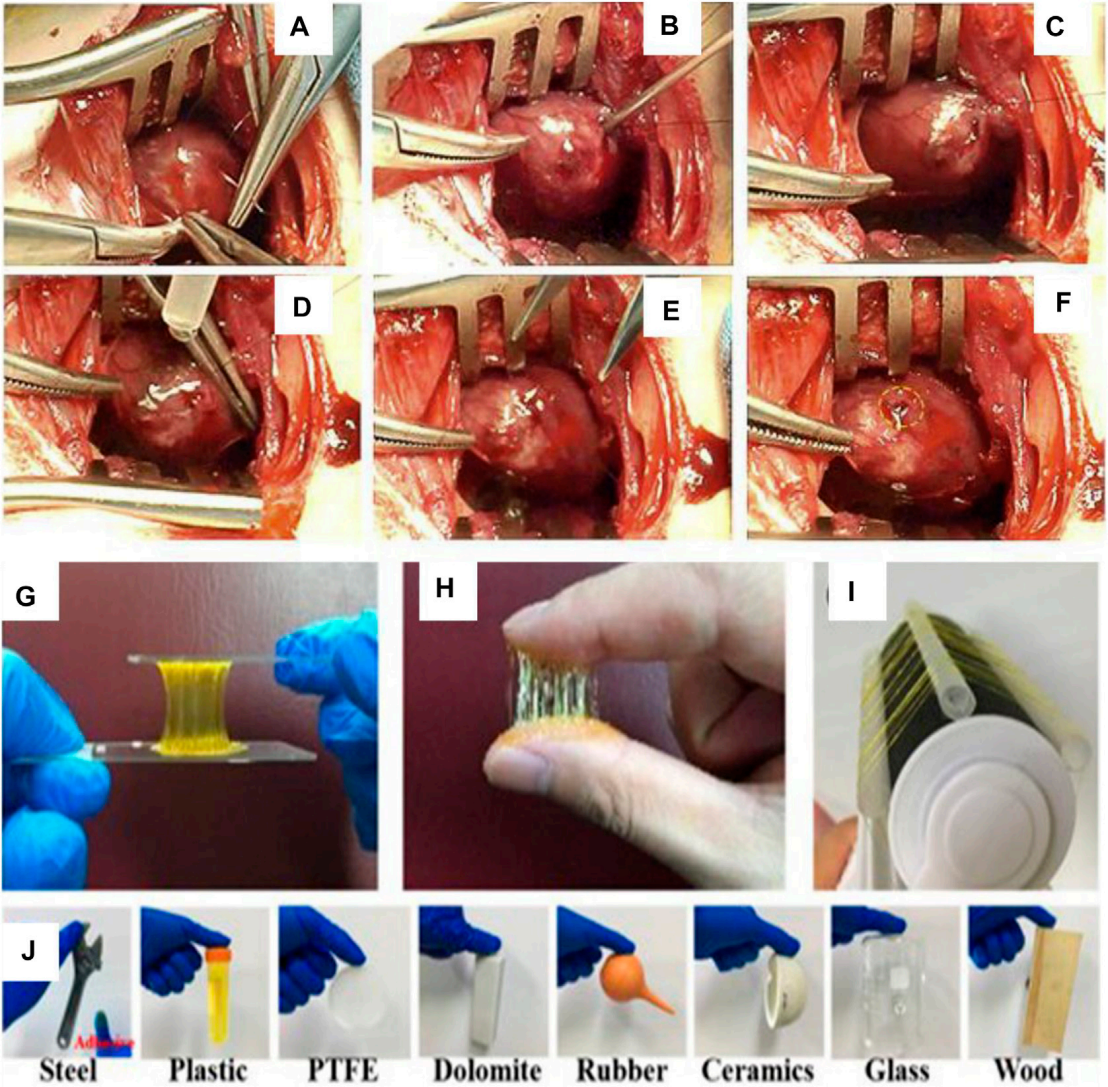
Polyethylene glycol/lysozyme hydrogel adheres to the defect site of the left ventricular wall in rabbits. **(A–F)** Hydrogel participates in the process of sealing the left ventricular defect (Tan et al., 2019). II. Adhesion experiments of aldehyde-functionalized hyaluronic acid/3,3′-dithiobis (propionyl hydrazide) hydrogels. **(G,J)** Hydrogels adhered to various substrate surfaces (Sigen et al., 2021).

At present, the tissue glue used in the clinical treatment of esophageal anastomotic fistula is mainly cyanoacrylate (superglue, highly toxic, and rarely used) and fibrin glue (fibrin glue, frequently used, but with low adhesion ability) (Rao et al., 2018). The adhesion strength and adhesion energy of fibrin glue are about 10 kPa and 10 Jm^−2^, respectively (Deng et al., 2019). As the esophagus is a soft tissue with peristalsis and swallowing functions, higher requirements are put forward for the glue used to bond the multi-layer esophageal scaffold (>>10 kPa and >>10 Jm^−2^). New adhesives, such as nano-clay/multi-walled carbon nanotubes/isopropylacrylamide hydrogel (adhesive strength 7 kPa) (Deng et al., 2019), sodium p-styrene sulfonate/chloromethane quaternized dimethylaminoethyl acrylate hydrogel (adhesive strength 25 kPa, adhesive energy 50 Jm^-2^) (Rao et al., 2018), chitosan/double-bonded phenylalanine hydrogel (adhesive strength 14 kPa) (Sharma et al., 2019), aldehyde functionalized hyaluronic acid/3,3′-dithiobis (propionyl hydrazide) hydrogel (adhesive strength 120 kPa) (Sigen et al., 2021), polyethylene glycol/lysozyme hydrogel (adhesive strength 32 kPa) (Tan et al., 2019), folic acid/polydimethyl diallyl ammonium chloride hydrogel (adhesive strength 150 kPa) (Gao et al., 2021), and hyaluronic acid/catechol/horseradish peroxidase hydrogels (17 kPa) (Wang D. et al., 2020) (Figure 7), are used. The hydrogels mentioned above can only meet one of the requirements of adhesion or cytocompatibility. Therefore, it is the research direction of adhesiveness of hydrogel to satisfy high adhesion and biocompatibility in a complex environment.

## Conclusion

Compared with traditional methods, esophageal tissue engineering technology has become a promising alternative method for the treatment of esophageal injury. The multi-layer complex structure of the esophagus should be considered in the repair of the full-thickness or circumferential defect of the esophagus, and how to obtain an ingenious design and retain the bionic structure and bionic function are the research direction. To solve these problems, the multi-step method is more favorable for the preparation of scaffolds; for example, glue bonding and 3D printing methods are two of the flexible styles to fabricate bionic scaffolds. It is believed that more and more perfect scaffolds will emerge in the near future and achieve more effective repair effects.
